# Physical Fitness, Exercise Behaviors, and Sense of Self-Efficacy Among College Students: A Descriptive Correlational Study

**DOI:** 10.3389/fpsyg.2022.932014

**Published:** 2022-07-15

**Authors:** Shan-shan Han, Bo Li, Guang-xu Wang, You-zhi Ke, Shu-qiao Meng, Ya-xing Li, Zhong-lei Cui, Wen-xia Tong

**Affiliations:** ^1^Institute of Sports Science, Nantong University, Nantong, China; ^2^School of Physical Education, Shanghai University of Sport, Shanghai, China; ^3^College of Physical Education, Henan Normal University, Xinxiang, China; ^4^Physical Education College, Yangzhou University, Yangzhou, China; ^5^Physical Education College, Shangqiu University, Shangqiu, China; ^6^Physical Education College, Shangqiu Normal University, Shangqiu, China

**Keywords:** physical fitness, exercise behavior, college students, self-efficacy, mediation effect

## Abstract

**Background:**

Self-efficacy is an important component of the mental well-being of college students. This study aimed to evaluate the development and the correlation between physical fitness (PF), exercise behavior, and self-efficacy in college students. To examine whether PF in individual college students can predict self-efficacy, and whether exercise behavior mediates this relationship.

**Methods:**

This was an observational study of 1923 randomly selected college students (50.5% girl). Measures included the Physical Activity Rating Scale, the Self-Efficacy Scale, and PF testing.

**Results:**

Self-efficacy was weakly correlated to both PF (*r* = 0.240) and exercise behavior (*r* = 0.248). In regression analysis, PF explained 24.7% of the variance in self-efficacy, increasing to 29.4% when exercise behavior was considered. Therefore, the predictive effect of PF on self-efficacy is partially realized through healthy exercise behavior.

**Conclusion:**

Physical fitness can predict self-efficacy among college students, with exercise behavior being an important mediation of this relationship. Strategies to improve positive exercise behaviors and PF could improve students’ self-efficacy and overall mental health.

## Introduction

The span of college education is a sensitive period for the psychological development of young adults, during which they develop coping strategies in terms of their mental health. The spread of the new coronavirus pneumonia (COVID-19) throughout the world has posed a great risk to human health. The COVID-19 pandemic and the resulting psychological problems pose an unprecedented health crisis and challenge for university students, who are a special group in the context of public health emergencies (Stowe et al., 2020). It has also been shown that people who are socially isolated have poorer cognitive skills, including memory and reaction time, and lower gray matter volume in many parts of the brain (Shen et al., 2022). Self-efficacy, the core concept of Bandura’s social cognition theory (Bandura, 1997), refers to the judgments and beliefs that individuals have regarding their ability to organize themselves to perform actions and achieve their goals. Self -efficacy has the following functions: (1) determine people’s choice of activities and the persistence of the activity; (2) affect people’s attitudes in front of difficulties; (3) affect the acquisition and performance of new behaviors; and (4) affect the emotions during the event (Bandura, 1997). The primary function of self-efficacy is behavioral regulation, which is manifested in the strength of an individual’s self-confidence (Bandura, 1997).

People with a high sense of self-efficacy are more likely to exert tremendous effort and perseverance to reach their goals. In comparison, individuals with a low sense of self-efficacy are more likely to have doubts about their abilities, to worry when they encounter failures and setbacks, and to generally be satisfied with mediocre achievements (Milne, 1999). Considering the recent evidence regarding the positive effects of physical exercise on mental health, improving individual self-efficacy through exercise has become an important means by which to promote the healthy development of individuals (Anderson and Feldman, 2020; Levy et al., 2020). Considering that some parts of China were in the period of the COVID-19 pandemic during the study period. Relevant evidence shows that high self-efficacy among college students during campus re-entry were only 50.4%, the level is low, respectively (Tadese and Mihretie, 2021). During the COVID-19 epidemic restraint management has a negative impact on mental health, about 40% college students experienced anxiety symptoms in China (Fu et al., 2021). And existing studies have shown that positive self-efficacy is closely related to the mental health of college students (Abdel-Khalek and Lester, 2017; Zeng et al., 2021), it is necessary to conduct special research on self-efficacy.

Physical fitness (PF) is a multifaceted construct involving physical and physiological components, including cardiorespiratory endurance, strength, speed, reaction, agility, balance, coordination, flexibility, etc. (Dong et al., 2019). PF is strongly associated with health (Xu et al., 2020). The Chinese government has always attached great importance to the PF of students. Since 2002, the *National Student Physical Health Standards* has been implemented, requiring all primary schools, middle schools and universities in China to conduct a *Student Physical Fitness Test* every year, and the test results are reported to the China Education department. The connotation of standard is an evaluation standard for measuring students’ physical health status and exercise effect. The main goals of the standard include three parts: education function, feedback function, and guiding students to do physical exercise (Ma et al., 2022). To this end, the relevant test indicators and results of the national student PF test can guide the development of Chinese students’ PF policy guidance (Xu et al., 2020).

We need to explore strategies to help students improve their sense of self-efficacy. Research on the self-efficacy of college students in China has shown that physical exercise exerts several positive effects, including improved happiness (Chen and Yu, 2015), self-concept and life satisfaction (Zeng and Zhao, 2007), and interpersonal trust and national identity (Han and Huang, 2019). Physical exercise is also an effective method to improve PF, which is an indicator of physical and mental health among college students. Extending from the existing research, this study aimed to evaluate the development of healthy PF, exercise behavior, and self-efficacy among college students and to explore the correlation between these constructs. The study of mediating variables can not only explain the mechanism behind the relationship, but also integrate existing research or theories, which has significant theoretical and practical significance. We hypothesized that there would be a positive relationship between exercise behavior and PF and self-efficacy in this population. PF in individual college students can predict self-efficacy, and exercise behavior mediates this relationship.

## Materials and Methods

### Study Group

We used a cluster sampling method to recruit first, second, and third-year undergraduate students from Nantong University, and Yangzhou University. Given that the senior year is at the stage of internship and dissertation (design) completion, this study did not sample seniors. All students provided informed consent before enrolling in the study. Students with previously diagnosed mental or physical health problems were excluded from the study group. The study protocol has been approved by the Ethics Committee at the Nantong University (NTU-ISS-2019-001).

The calculation of the minimum sample size is completed by Equation 1 (Shao, 2012), where the type I error α is set to 0.05, the allowable error δ is set to 0.01, and the sample rate ρ is set to 0.05. A total of 54,689 people (data updated in 2019), so the limited overall number *N* sets 75% of the total number of students to be about 41,017. The minimum sample size required for this study was calculated to be 1,748.


(1) $$n=\frac{{\left(\frac{{\text{Z}}_{\alpha }}{\delta }\right)}^{{}^{2}}\times \rho \times \left(1-\rho \right)}{1+\left[{\left(\frac{{\text{Z}}_{\delta }}{\delta }\right)}^{2}\times \rho \times \left(1-\rho \right)\right]/\text{N}}$$

In all, 2,188 students were randomly selected and they completed our PF test. After the test, students were asked to complete a questionnaire regarding their physical activity and self-efficacy. Of the 2,188 students who completed the PF test, 2,094 returned their questionnaires. Of these, 1,923 questionnaires were complete (effective response rate, 91.8%), and those individuals formed our study sample, comprising 951 men and 972 women. The characteristics of the study sample are summarized in Table 1. The data obtained were verified against student identification to match physical test results and completed questionnaires.

**TABLE 1:** Characteristics of the study sample.

	Male	Female	Total	Proportion (%)
First-year college students	211	215	426	22.2
Second-year college students	424	499	923	48.0
Third-year college students	316	258	574	29.8
Total	951	972	1,923	100.0

### Testing Method

#### Physical Activity Rating Scale

The physical exercise rating scale was compiled by the Japanese scholar Takao Hashimoto, which was subsequently introduced and completed by Liang (1994) in China. Physical exercise volume was examined with respect to the exercise intensity, frequency, and time of one exercise activity to measure physical exercise participation.


(2) Physical⁢exercise⁢volume⁢score=intensity×(time-1)×frequency

Each parameter was evaluated using five score levels. The level standards were as follows: small exercise volume ≤ 19 points, moderate exercise volume = 20–42 points, and large exercise amount ≥43 points (Liang, 1994). The re-test reliability of this scale was 0.820. Follow-up related research shows that the internal consistency reliability of Physical Activity Rating Scale is Cronbach’s α = 0.85. The value of the scale was used to reflect the physical exercise behaviors of college students (Collins et al., 2022).

#### General Self-Efficacy Scale

Self-efficacy was evaluated using the Chinese version of the General Self-Efficacy Scale developed by Luszczynska et al. (2005). This scale is appropriate to use for individuals ≥12 years of age. The reliability and validity of this scale have previously been reported in a Chinese population. The scale has good reliability, with a Cronbach’s α of 0.87, test-retest reliability correlation (r) of 0.83 (p < 0.001), and half-reliability agreement (r) of 0.82 (n = 401, p < 0.001) (Wang and Liu, 2001). The 10 items of this scale were scored on a 4-point Likert scale, with higher scores indicative of higher self-efficacy.

#### Physical Fitness Test

Physical fitness levels were quantified in a physical fitness index (PFI) using The National Student Physical Health Standards (Revised in 2014) of the Chinese Student PF Standards. The student fitness tester (Model: HK6800; Shenzhen Hengkang Jiaye Technology Co., Ltd.) was used to measure the following components of PF. The PF test was performed as part of the college physical education curriculum. Before the test, students needed to perform a standardized warm-up for 5 min, including muscle stretching and joint exercises. According to the curriculum arrangement of the test school, college physical education is generally arranged between 9:00 and 11:00 in the morning and 2:00 and 5:00 in the afternoon, and the physical education class is only available under clear and conducive weather and temperature conditions.

The test was conducted in the physical education class, enabling suitable temperature and humidity during the test. Participants completed each test item once per day for three days, and the best score of the three was used in the analysis. After participants had completed the physical tests, they were asked to complete the questionnaire on physical activity and self-efficacy. The tests and questionnaires were completed during March–April, 2020. After we have collected the data, we need to calculate the physique score of college students according to the norm of The National Student Physical Health Standards (Revised in 2014). For the conversion norm, refer to Supplementary Appendix 1. The main test procedures are as follows.

(1) Body shape: test the body mass index (BMI, kg/m^2^).

Standing height barefoot will be measured using a stable stadiometer (GMCS-SGZG3, Jian-Min, Beijing) to the nearest 0.001 m. Bodyweight with light clothes will be measured using a portable scale (GMCS-YERCS3, Jian-Min, Beijing) to the nearest 0.1 kilograms (kg). BMI was calculated by height and body weight:


$$\text{BMI}\,\left(\text{kg}/m^{2}\right)=\frac{\text{Weight}\,\left(\text{kg}\right)}{{\left(\text{Height}\right)}^{2}\,\left({\text{m}}^{2}\right)}$$

(2) Cardiorespiratory endurance: the test items were time required to run distances of 1,000 m for boys and 800 m for girls.

The test equipment includes 400, 300, and 200 m orbiting tracks, and the geology is not limited. Other irregular venues can also be used, but the measurement must be accurate and the venue flat. Several stopwatches need to be corrected before use, and the error per min should not exceed 0.2 s. The standard stopwatch is selected based on Beijing time, with an error of no more than 0.3 s per h. At least two subjects will be tested in pairs, commencing from a standing start point. Start running after hearing the “Run” command. The timekeeper will see the flag to start the watch, and stop the watch when the subject’s torso reaches the vertical plane of the finish line. Record the test results in seconds, accurate to one digit after the decimal point, and the second digit after the decimal point is considered according to the principle of non-zero advancement: for example, 10.11 s as 10.2 s.

(3) Flexibility, measured using the seated forward flexion test.

Before the test, the subject should prepare a preparation to prevent zooming. The subject is sitting on the cushion connected to the box. The legs are straightened and should not be bent. The heels are close together. The toes are separated by about 10–15 cm. The two arms and hands are straight, gradually make the upper body to flex, gently push the cursor on the rib in both hands (not suddenly reaching it) until it continues to extend before extending. The inner plane of the pedal of the test score is 0 points, the inward is negative, and the forward is positive. Record in the units of centimeters, take a decimal point. If it is a positive value, add the “+” symbol before the value, and the negative value will be added with the “−” symbol.

(4) Power, measured using the standing long jump test.

The subject stands separately on both feet. After standing behind the starting line, the toes must not be stepped on (it is best to use a wire rope to start the line). Both feet are jumping at the same time, and there must be no steps or jumping movements. Measure the vertical distance from the rear edge of the jump line to the rear edge of the nearest place. Each person trial three times and record the best results. In the unity of centimeters, regardless of decimal.

### Data Analysis

All analyses were performed using IBM SPSS (version 25.0). Step 1: the association between PF, exercise behavior and self-efficacy was evaluated using Pearson’s or Spearman’s rank order correlation, as appropriate for the data distribution. Step 2: a linear regression analysis was used to evaluate the specific association between an individual’s exercise behavior and the components of PF measured (body shape, cardiorespiratory endurance, flexibility, and power). Step 3: a hierarchical regression analysis (Model A) was used to evaluate the predictive effect of each component of PF (independent variables) on self-efficacy (dependent variable). In the Model B regression, exercise behavior was also included as an independent variable. Steps 1 and 2 together constitute the methodology of stepwise regression analysis, which is the most commonly used method for testing mediation effects (MacKinnon et al., 2007).

## Results

The descriptive data (mean and SD) of the participants according to course and gender in the dependent variables in Table 2.

**TABLE 2:** List of descriptive data (N = 1,923).

		Grade						Gender			
	Total		First		Second		Third		Male		Female	
	M	SD	M	SD	M	SD	M	SD	M	SD	M	SD
**Physical fitness**												
F 1 body shape	94.380	11.047	92.676	12.228	95.385	10.201	94.042	11.273	91.483	13.083	97.222	7.601
F 2 cardiorespiratory endurance	79.650	14.915	78.897	18.048	81.410	13.571	77.371	14.059	79.303	14.931	79.985	14.900
F 3 flexibility	75.660	16.478	75.883	17.796	75.996	16.011	74.939	16.205	71.953	17.152	79.278	14.935
F 4 power	66.072	17.058	65.023	16.967	67.035	16.880	65.301	17.353	62.921	19.342	69.154	13.811
F all physical fitness	71.926	9.440	72.009	9.390	72.637	9.609	70.723	9.091	66.107	8.421	77.621	6.432
**Exercise behavior**												
E 1 intensity	2.471	1.192	2.535	1.229	2.427	1.164	2.505	1.206	2.782	1.124	2.173	1.180
E 2 length	3.472	1.096	3.462	1.129	3.463	1.073	3.476	1.109	3.628	1.104	3.309	1.065
E 3 frequency	3.120	0.817	3.162	0.846	3.103	0.805	3.132	0.816	3.101	0.768	3.148	0.863
E all exercise behavior	21.250	17.684	21.587	18.260	20.824	17.275	21.672	17.913	25.429	18.872	17.154	15.383
**Self-efficacy**												
Self-efficacy	36.990	9.768	37.354	9.321	36.809	10.013	37.012	9.702	38.265	9.908	35.744	9.470

### Correlation Analysis

The outcomes of the correlation analysis between PF, exercise behavior, and self-efficacy are summarized in Table 3. Self-efficacy was weakly correlated with PF (*r* = 0.240) and exercise behavior (*r* = 0.248). Nonetheless, the correlation between students’ self-efficacy and the components of PF and exercise behavior was positive and significant (*p* < 0.05 and *p* < 0.01, respectively).

**TABLE 3:** Correlation between physical fitness, exercise behavior, and self-efficacy (N = 1,923).

	F 1	F 2	F 3	F 4	F all	E 1	E 2	E 3	E all
**Physical fitness**									
**F 1 body shape**									
F 2 cardiorespiratory endurance	0.067**								
F 3 flexibility	0.160**	0.175**							
F 4 power	0.360**	0.274**	0.245**						
F all physical fitness	0.575**	0.502**	0.677**	0.778**					
**Exercise behavior**									
E 1 intensity	−0.009	0.040*	0.057**	0.128**	0.079**				
E 2 length	−0.023	0.050**	−0.001	0.079**	0.037*	0.422**			
E 3 frequency	−0.008	0.044*	0.055**	0.043*	0.046**	0.068**	0.150**		
E all exercise behavior	−.0540*	0.083**	0.083**	0.111**	0.097**	0.689**	0.683**	0.289**	
**Self-efficacy**									
Self-efficacy	−0.035	0.451*	0.127**	0.263**	0.240**	0.250**	0.325**	0.107**	0.248**

*p < 0.05, **p < 0.01.

### Regression Analysis of Exercise Behavior and Physical Fitness

Results of the regression analysis are reported in Table 4. Exercise behavior exerted a significant effect (*p* < 0.05) on the components of PF, except body shape exercise intensity, duration, and frequency were all predictors of PF. Exercise intensity was specifically associated with two components of PF: flexibility (β = 0.083) and power (β = 0.084). Exercise duration was associated with cardiopulmonary fitness (β = 0.054) and power (β = 0.057), with exercise frequency being associated with flexibility (β = 0.026) and power (β = 0.030). Each sub-indicator of exercise behavior had a predictive effect on the total score for PF.

**TABLE 4:** Regression analysis between exercise behavior and physical fitness (*N* = 1,923).

Dependent/independent	Body shape		Cardiorespiratory endurance		Flexibility		Power		Healthy fitness	
	β	*t*	β	*t*	β	*t*	β	*t*	β	*t*
Intensity	0.013	0.500	0.032	1.264	0.083	3.321*	0.084	3.372**	0.091	3.654**
Duration	−0.020	−0.783	0.054	2.134**	0.004	0.166	0.057	2.269*	0.038	1.494*
Frequency	−0.024	−1.023	0.044	1.900	0.026	1.145*	0.030	1.327*	0.031	1.360*
*F*	0.651		5.216		5.252		10.432		9.218	
*R* ^2^	0.001		0.018**		0.018**		0.026**		0.024**	

*p < 0.05, **p < 0.01.

### Hierarchical Multiple Regression Analysis of Physical Fitness, Self-Efficacy, and Exercise Behavior

As shown in Table 5, both regression Models A and B were significant (*p* < 0.01). In Model A, with self-efficacy as the dependent variable and PF as the independent variable, all components of PF exerted a strong predictive effect on self-efficacy. Model B, in which PF and exercise behavior were considered simultaneously, increased the variance accounted for in Model A (*R*^2^-value increase from 0.247 to 0.294). Therefore, exercise behavior exerted a significant impact on the relationship between PF and self-efficacy.

**TABLE 5:** Hierarchical multiple regression analysis of physical fitness, self-efficacy, and exercise behavior (*N* = 1,923).

Dependent/independent	Model A					Model B				
	β	t	*p*	95% CI		β	t	*p*	95% CI	
Body shape	–0.116	–4.855	<0.001	–0.145	–0.061	–0.100	–4.278	<0.001	–0.129	–0.048
Cardiorespiratory endurance	–0.010	–0.421	0.674	–0.057	0.037	–0.020	–0.892	0.372	–0.066	0.025
Flexibility	0.103	4.426	<0.001	0.034	0.088	0.090	3.977	<0.001	0.027	0.080
Power	0.183	7.267	<0.001	0.076	0.133	0.156	6.304	<0.001	0.061	0.117
Intensity						0.138	5.709	<0.001	0.740	1.514
Duration						0.083	3.398	0.001	0.311	1.160
Frequency						0.099	4.492	<0.001	0.667	1.700
*F*	23.401**					28.364**				
*R* ^2^	0.247					0.294				
Δ*R*^2^	0.245					0.291				

**p < 0.01.

## Discussion

Our results show that PF predicted self-efficacy overall, with a further significant predictive effect of the components of PF on self-efficacy. Overall, PF explained 24.7% of the variance in self-efficacy. When we added physical exercise behavior as an intermediary variable, the fit of the regression model improved, the variance explained went from 24.7 to 29.4%. This finding underlines the importance of exercise behavior for PF and ultimately, self-efficacy. Of note is that when exercise behavior was included, only BMI, flexibility, and power were retained as predictors of self-efficacy. In addition to the above results, this study also found that college students had lower PF overall, with power being the worst.

### Power Is the Worst Physical Fitness Among Them

This result is similar to other studies (Cui et al., 2018; Wang, 2019; Li et al., 2022). The college student population is a reserve of high-quality national talent. However, the decline in the PF of college students is of great concern. From 1985 to 2014, 1,513,435 students participated in the Chinese National Survey on Student Constitution and Health. A decline in the PFI was observed between 1985 and 2014 (overall PFI change of 0.8), albeit with an increase from 1985 to 1995 (PFI change of 1.2) (Dong et al., 2019). This confirms the decline in the PF of college students. There are many reasons for this phenomenon, with existing research focusing on the overall decline in physical activity among college students and the increase in sedentary time. World Health Organization released a new version of the Guidelines on Physical Activity and Sedentary Behavior, reminding adults (aged 15–64 years) to engage in 150–300 min of moderate-intensity or 75–150 min of high-intensity activity every day (Bull et al., 2020). The latest statistics show that one-quarter of adults and four-fifths of adolescents are not meeting these physical activity guidelines (Guthold et al., 2018).

For the time period in which the data was collected in this study, the decline in physical activity among college students appears to be more severe due to the impact of COVID-19. In order to cut off the spread of COVID-19, Chinese local governments have implemented related confinement policies, which is the main reason for the decline in physical activity. The resulting negative phenomenon of human mental health is also very clear. A recent study published in Neurology suggests that social isolation is associated with reduced brain volume in cognitively relevant areas and an increased risk of dementia. Its results suggest that unlike risk factors such as depression and loneliness, social isolation is associated with a 26% increased risk of dementia (Shen et al., 2022). Students whose family residence was worst hit by the pandemic tend to report poorer mental health during the pandemic outbreak, risk perception of being infected with COVID-19 has a negative direct and indirect impacts on college students’ mental health (Han et al., 2021). During the COVID-19 epidemic restraint management has a negative impact on mental health, more than 40% college students experienced anxiety symptoms in China (Fu et al., 2021). Another study showed that of participants, 32.5% reported that COVID-19 negatively impacted their stress levels very much or an extreme amount, 29.0% reported a moderate negative impact and only 38.5% reported little to no negative impacts (Prowse et al., 2021).

### Physical Fitness Can Effectively Predict the Self-Efficacy of College Students

According to the analysis, college students with better PF will also have higher overall self-evaluation and evaluation of specific aspects of the body. In other words, college students with higher PF have higher body self-esteem (Xie, 2012). Self-confidence in one’s own body also extends to other areas. When encountering different environments, facing new environments or encountering difficult problems, self-confidence in adapting to the environment and solving problems will also increase, that is self-efficacy increases. However, the results of this study show exercise behavior does not predict weight status. This is quite different from previous studies (Zhang et al., 2020). The possible reason is that the body shape used in this study is the norm for BMI of college students in A, which is only developed based on the situation of Chinese college students and the norm is relatively old.

### Exercise Behavior Has Strong Predictive Effect on Self-Efficacy, With a Demonstrated Improvement in Individual Self-Confidence in Exercise

Our findings revealed a high degree of positive correlation between self-efficacy and exercise behavior. This is consistent with previously reported findings, which state that the more active exercise behavior, the higher the self-efficacy (Anderson and Feldman, 2020). Self-efficacy is considered an important source of internal motivation for individuals to participate in exercise. Moreover, self-efficacy among college students can also play a significant role in predicting exercise activity (Yu et al., 2013). Evidence also points to a significant positive correlation between the self-efficacy of participating in exercise and motivational readiness, walking steps, and calorie consumption (Barr-Anderson et al., 2007). Therefore, improving college students’ self-efficacy to participate in exercise can increase the frequency of students’ participation in exercise, leading to improvement in their PF status (Annesi, 2006).

### Exercise Behavior Is an Important Mediating Variable Between Physical Fitness and Self-Efficacy

The purpose of studying the mediating effect is to explore the internal mechanism of the relationship based on the known relationship between PF and self-efficacy. In this process, the original research on the same phenomenon can be linked together, the theories originally used to explain similar phenomena can be integrated, and the existing theories can be made more systematic (Preacher et al., 2007). The establishment table of the mediation model for the three variables of PF, exercise behavior and self-efficacy, there may be a “Matthew effect” among the three. Specifically, when a college student’s PF is better, he has a more active exercise behavior and thus has a higher self-efficacy. The reverse is also true. PF in individual college students can predict self-efficacy, and exercise behavior mediates this relationship. This will provide reference for scholars who are engaged in the research of college students’ mental health.

The limitations of our study should be acknowledged when interpreting the results. First, both exercise and self-efficacy involve multiple dimensions, and the mechanisms may differ across dimensions. Second, we only considered the association between exercise and self-efficacy. In reality, self-efficacy may be influenced by other variables; therefore, the relationship between exercise and self-efficacy may be more complex than shown in this study.

## Conclusion

Physical fitness can improve self-efficacy among college students, with exercise behavior being an important mediator to this relationship. Our findings provide a theoretical basis for developing strategies to improve the self-efficacy and overall mental health of college students by promoting positive exercise behaviors and PF.

## Data Availability Statement

The original contributions presented in this study are included in the article/Supplementary Material, further inquiries can be directed to the corresponding author.

## Ethics Statement

The studies involving human participants were reviewed and approved by the Nantong University. The patients/participants provided their written informed consent to participate in this study. Written informed consent was obtained from the individual(s) for the publication of any potentially identifiable images or data included in this article.

## Author Contributions

S-SH, BL, Y-ZK, G-XW, S-QM, and Y-XL wrote the main manuscript text. Z-LC and W-XT wrote the statistical analysis and tables. All authors reviewed the manuscript.

## Conflict of Interest

The authors declare that the research was conducted in the absence of any commercial or financial relationships that could be construed as a potential conflict of interest.

## Publisher’s Note

All claims expressed in this article are solely those of the authors and do not necessarily represent those of their affiliated organizations, or those of the publisher, the editors and the reviewers. Any product that may be evaluated in this article, or claim that may be made by its manufacturer, is not guaranteed or endorsed by the publisher.

## References

[B1] Abdel-Khalek A. M. Lester D. (2017). The association between religiosity, generalized self-efficacy, mental health, and happiness in Arab college students. *Pers. Individ. Diff.* 109 12–16. 10.1016/j.paid.2016.12.010

[B2] Anderson C. L. Feldman D. B. (2020). Hope and Physical Exercise: The Contributions of Hope, Self-Efficacy, and Optimism in Accounting for Variance in Exercise Frequency. *Psychol. Rep.* 123 1145–1159. 10.1177/0033294119851798 31142190

[B3] Annesi J. J. (2006). Relations of physical self-concept and self-efficacy with frequency of voluntary physical activity in preadolescents: implications for after-school care programming. *J. Psychosom. Res.* 61 515–520. 10.1016/j.jpsychores.2006.04.009 17011360

[B4] Bandura A. (1997). *Self-Efficacy: the Exercise of Control.* New York, NY: W. H. Freeman and Co.

[B5] Barr-Anderson D. J. Young D. R. Sallis J. F. Neumark-Sztainer D. R. Gittelsohn J. Webber L. (2007). Structured physical activity and psychosocial correlates in middle-school girls. *Prev. Med.* 44 404–409. 10.1016/j.ypmed.2007.02.012 17363050

[B6] Bull F. C. Al-Ansari S. S. Biddle S. Borodulin K. Willumsen J. F. (2020). World Health Organization 2020 guidelines on physical activity and sedentary behaviour. *Br. J. Sports Med.* 54 1451–1462. 33239350 10.1136/bjsports-2020-102955 PMC7719906

[B7] Chen Z.-Y. Yu P. (2015). The effects of physical education on the subjective well-being of college students: the intermediate effect of companion. *J. Capital Univ. Phys. Educ. Sport* 27 165–171.

[B8] Collins H. M. Fawkner S. Booth J. N. Duncan A. (2022). The impact of resistance training on strength and correlates of physical activity in youth. *J. Sports Sci.* 40 40–49. 10.1080/02640414.2021.1976487 34533102

[B9] Cui Y. F. Zhang W. B. Gong Q. Chen Y. B. Chen S. L. Wu Z. Q. (2018). Frequency of Breakfast and Physical Fitness among Chinese College Students. *Am. J. Health Behav.* 42 156–162. 10.5993/AJHB.42.1.15 29320348

[B10] Dong Y. Lau P. W. C. Dong B. Zou Z. Yang Y. Wen B. (2019). Trends in physical fitness, growth, and nutritional status of Chinese children and adolescents: a retrospective analysis of 1.5 million students from six successive national surveys between 1985 and 2014. *Lancet Child Adolesc. Health* 3 871–880. 10.1016/S2352-4642(19)30302-5 31582349

[B11] Fu W. Yan S. Zong Q. Anderson-Luxford D. Song X. Lv Z. (2021). Mental health of college students during the COVID-19 epidemic in China. *J. Affect. Disord.* 280 7–10. 10.1016/j.jad.2020.11.032 33197782 PMC7656159

[B12] Guthold R. Stevens G. A. Riley L. M. Bull F. C. (2018). Worldwide trends in insufficient physical activity from 2001 to 2016: a pooled analysis of 358 population-based surveys with 1.9 million participants. *Lancet Glob. Health* 4 23–35. 10.1016/S2214-109X(18)30357-7 30193830

[B13] Han H.-Y. Huang J.-Y. (2019). Review of the social psychology function of physical education. *J. Shandong Coll. Phys. Educ.* 35 13–19.

[B14] Han Z. Tang X. Li X. Shen Y. Li L. Wang J. (2021). COVID-19-Related Stressors and Mental Health Among Chinese College Students: A Moderated Mediation Model. *Front. Public Health* 9:586062. 10.3389/fpubh.2021.586062 34222162 PMC8253361

[B15] Levy S. S. Thralls K. J. Goble D. J. Krippes T. B. (2020). Effects of a Community-Based Exercise Program on Older Adults’ Physical Function, Activities of Daily Living, and Exercise Self-Efficacy: Feeling Fit Club. *J. Appl. Gerontol.* 39 40–49. 10.1177/0733464818760237 29504440

[B16] Li B. Han S. S. Meng S. Q. Lee J. Cheng J. Liu Y. (2022). Promoting exercise behavior and cardiorespiratory fitness among college students based on the motivation theory. *BMC Public Health* 22:738. 10.1186/s12889-022-13159-z 35418043 PMC9009046

[B17] Liang D. (1994). Stress levels of college students and their relationship to physical exercise. *Chin. Ment. Health J.* 8 5–6.

[B18] Luszczynska A. Scholz U. Schwarzer R. (2005). The General Self-Efficacy Scale: Multicultural Validation Studies. *J. Psychol.* 139 439–457. 16285214 10.3200/JRLP.139.5.439-457

[B19] Ma N. Dang J. Liu Y. Zhong P. Yan X. Zhang J. (2022). Percentile Curves for Multiple Physical Fitness Components Among Chinese Han Children and Adolescents Aged 7-18 Years From a National Survey Based on the Total and the Normal Weight Population. *Front. Nutr.* 8:770349. 10.3389/fnut.2021.770349 35047541 PMC8762235

[B20] MacKinnon D. P. Fairchild A. J. Fritz M. S. (2007). Mediation analysis. *Annu. Rev. Psychol.* 58 593–614. 10.1146/annurev.psych.58.110405.085542 16968208 PMC2819368

[B21] Milne S. E. (1999). Self-efficacy in changing societies. *J. Health Psychol.* 4 281–283. 10.1177/135910539900400207 22021491

[B22] Preacher K. J. Rucker D. D. Hayes A. F. (2007). Addressing Moderated Mediation Hypotheses: Theory, Methods, and Prescriptions. *Multivar. Behav. Res.* 42 185–227. 10.1080/00273170701341316 26821081

[B23] Prowse R. Sherratt F. Abizaid A. Gabrys R. L. Hellemans K. G. C. Patterson Z. R. (2021). Coping With the COVID-19 Pandemic: Examining Gender Differences in Stress and Mental Health Among University Students. *Front. Psychiatry* 12:650759. 10.3389/fpsyt.2021.650759 33897499 PMC8058407

[B24] Shao Z.-Q. (2012). How to Determine the Sample Size in Sampling Survey. *Stat. Decis.* 22 12–14. 10.13546/j.cnki.tjyjc.2012.22.002

[B25] Shen C. Rolls E. Cheng W. Kang J. Dong G. Xie C. (2022). Associations of Social Isolation and Loneliness With Later Dementia. *Neurology* 99 1–12. 10.1212/wnl.0000000000200583 35676089

[B26] Stowe R. C. Smidt S. D. E. Mason T. A. (2020). Emerging Subspecialties in Neurology: sleep medicine fellowship after child neurology residency. *Neurology* 94 278–281. 10.1212/WNL.0000000000008759 31992679

[B27] Tadese M. Mihretie A. (2021). Attitude, preparedness, and perceived self-efficacy in controlling COVID-19 pandemics and associated factors among university students during school reopening. *PLoS One* 16:e0255121. 10.1371/journal.pone.0255121 34473719 PMC8412257

[B28] Wang C. Liu Y. (2001). Reliability and validity of the General Self-Efficacy Scale. *Chin. J. Appl. Psychol.* 7 37–40.

[B29] Wang J. L. (2019). The association between physical fitness and physical activity among Chinese college students. *J. Am. Coll. Health* 67 602–609. 10.1080/07448481.2018.1515747 30849024

[B30] Xie Q.-W. (2012). Relationship among Physical Exercise, Physical Self-esteem and General Self-efficacy of Undergratuates. *J. Guangzhou Sport Univ.* 32 95–99.

[B31] Xu R. Song Y. Hu P. Dong B. Zou Z. Luo D. (2020). Towards Comprehensive National Surveillance for Adolescent Health in China: Priority Indicators and Current Data Gaps. *J. Adolesc. Health* 67 14–23. 10.1016/j.jadohealth.2020.05.043 33246529

[B32] Yu L. Xia J.-M. Zhang W.-W. (2013). Effects of physical activity intervention on mental health and self-efficacy of students with weak physical fitness. *J. Wuhan Inst. Phys. Educ.* 47 74–77. 10.1186/s12913-016-1423-5 27409075 PMC4943498

[B33] Zeng Q. Zhao D-l (2007). Effect of physical exercise on adolescent body self-concept and life satisfaction. *J. Wuhan Inst. Phys. Educ.* 41 59–63.

[B34] Zeng Y. Qiu S. P. Alizadeh A. Liu T. F. (2021). How Challenge Stress Affects Mental Health among College Students during the COVID-19 Pandemic: The Moderating Role of Self-Efficacy. *Int. J. Ment. Health Promot.* 23 167–175. 10.32604/IJMHP.2021.015937

[B35] Zhang X. Chen S. Gu X. (2020). Movement behaviors and health-related fitness among pubertal adolescents: 2012 NHANES national youth fitness survey data. *J. Sports Med. Phys. Fit.* 61 983–990. 10.23736/S0022-4707.20.11527-5 33146498

