# Supplementary material for: Physical Fitness, Exercise Behaviors, and Sense of Self-Efficacy Among College Students: A Descriptive Correlational Study
Source: Front Psychol. 2022 Jul 15;13:932014. doi: 10.3389/fpsyg.2022.932014 (PMC9335150; doi:10.3389/fpsyg.2022.932014)
Supplement: Supplementary file 1 [file Data_Sheet_1.doc]

**National Student Physical Health Standards（Revised in 2014）**

# Description

1. The "National Student Physical Health Standard" (hereinafter referred to as the "Standard") is the basic guiding document and the basic standard of education quality for the national school education work. It is the specific implementation of the "National Physical Exercise Standards" in schools, and is suitable for students in full-time ordinary primary schools, junior high schools, ordinary high schools, secondary vocational schools, and ordinary institutions of higher learning.

2. The revision of this standard adheres to health first, and implements the "Outline of the National Medium- and Long-Term Education Reform and Development Plan (2010-2020)", "The General Office of the State Council forwards the notice of the Ministry of Education and other departments on further strengthening the school sports work". (Guobanfa [2012] No. 53) and the "Ministry of Education Notice on Printing and Distributing Three Documents including the "Measures for the Monitoring and Evaluation of Students' Physical Health" (Jiao Ti-yi [2014] No. 3), focusing on improving the application of the "Standard" It focuses on strengthening its functions of educational incentives, feedback adjustment and guiding exercise, and focuses on improving its educational monitoring and performance evaluation support capabilities.

3. This standard comprehensively evaluates the physical health level of students from the aspects of body shape, physical function and physical quality. It is an educational means to promote the healthy development of students' physical fitness and encourage students to actively exercise. It is the national core quality system for student development and academic quality. An important part of the standard is the individual evaluation standard of students' physical health.

4. This standard divides the applicable objects into the following groups: primary school, junior high school, and high school are divided into one group for each grade, including 6 groups for elementary school, 3 groups for junior high school, and 3 groups for high school. The first and second grades are a group, and the third and fourth grades are a group.

5. The test indicators for each group of elementary school, junior high school, high school and university are mandatory indicators. Among them, the height and weight in the body shape category, the vital capacity in the physical function category, and the 50-meter running and sitting forward flexion in the physical fitness category are the common indicators for students of all grades.

6. The total score for this standard school year is composed of the sum of the standard score and the additional score, and the full score is 120 points. The standard score is composed of the sum of the product of each individual indicator score and the weight, and the full score is 100 points. The additional points are determined according to the actual measured results, that is, the extra points for the extra points that exceed 100 points are added, and the full score is 20 points; the extra points for elementary school are skipping rope for 1 minute, and the extra points are 20 points; the extra points for junior high school, high school and university The sub-indicators are pull-ups and 1,000-meter run for boys, and 1-minute sit-ups and 800-meter run for girls. The bonus points for each index are 10 points.

7. According to the total score of the student in the academic year, the grade is: 90.0 points and above are excellent, 80.0-89.9 points are good, 60.0-79.9 points are passing, and 59.9 points and below are failing.

8. Each student will be assessed once every academic year and recorded in the Registration Card of the "National Student Physical Health Standards" (Appendix 1 to 6). For schools with special educational system, when filling out the registration card, the columns can be increased or decreased accordingly according to regulations and needs. The grades and grades of students upon graduation shall be assessed based on the sum of 50% of the total marks of the academic year in which they graduated and 50% of the average marks of other academic years.

9. Students who have achieved good or above in the test scores can participate in the evaluation and awards; only those who have achieved excellent scores can receive credits for the sports award. Those who fail the test scores will be allowed to take a supplementary test in the current academic year. If the supplementary test still fails, the school year will be assessed as a failure. When students graduate from ordinary high schools, secondary vocational schools and ordinary institutions of higher learning, those with a score of less than 50 in the "Standard" test will be considered as completion or dropout.

10. Due to illness or disability, students can submit an application to the school for suspension or exemption from the implementation of the "Standards". With the certification of the medical unit and the approval of the physical education department, the implementation of the "Standards" can be suspended or exempted, and fill in the "Exemption from Implementation <National Student Physical Fitness". Health Standards > Application Form" (Schedule 7), which is stored in the student file. Students with disabilities who have indeed lost their athletic ability and are exempted from the implementation of the "Standards" can still participate in the evaluation and awards.

11. Every school year, each school carries out the "Standard" test covering students of all grades in the school. After the "Standard" test data is reviewed by the local education administrative department as required, it is uploaded to the "National Student Physical Health Standard Data Management" through the "Chinese Student Physical Health Network". system". Testing and data upload times are determined by the Education Administration.

12. This standard is interpreted by the Ministry of Education.

# Individual indicators and weights

| indicator | Weights（%） |
| --- | --- |
| BMI | 15 |
| lung capacity | 15 |
| 50m run | 20 |
| Sitting forward bend | 10 |
| Standing long jump | 10 |
| Pull-ups (men) / 1 minute sit-ups (women)  坐（女） | 10 |
| 1000m run (men)/800m run (women) | 20 |

# Evaluation form

**Table 1-1 Male body mass index (BMI) score table (kg/m^2^)**

| **Grade** | **single score** | **interval** |
| --- | --- | --- |
| normal | **100** | 17.9~23.9 |
| low body weight | **80** | ≤17.8 |
| overweight |  | 24.0~27.9 |
| obesity | **60** | ≥28.0 |

**Table 1-1 Female body mass index (BMI) score table (kg/m^2^)**

| **Grade** | **single score** | **interval** |
| --- | --- | --- |
| normal | **100** | 17.2~23.9 |
| low body weight | **80** | ≤17.1 |
| overweight |  | 24.0~27.9 |
| obesity | **60** | ≥28.0 |

**Table 1-3 Males' lung capacity single item score table (ml)**

| **Grade** | **single score** | **Freshman and Sophomore** | **Junior and Senior** |
| --- | --- | --- | --- |
| excellent | **100** | 5040 | 5140 |
|  | **95** | 4920 | 5020 |
|  | **90** | 4800 | 4900 |
| good | **85** | 4550 | 4650 |
|  | **80** | 4300 | 4400 |
| Pass | **78** | 4180 | 4280 |
|  | **76** | 4060 | 4160 |
|  | **74** | 3940 | 4040 |
|  | **72** | 3820 | 3920 |
|  | **70** | 3700 | 3800 |
|  | **68** | 3580 | 3680 |
|  | **66** | 3460 | 3560 |
|  | **64** | 3340 | 3440 |
|  | **62** | 3220 | 3320 |
|  | **60** | 3100 | 3200 |
| failed | **50** | 2940 | 3030 |
|  | **40** | 2780 | 2860 |
|  | **30** | 2620 | 2690 |
|  | **20** | 2460 | 2520 |
|  | **10** | 2300 | 2350 |

**Table 1-4 Females' lung capacity single item score table (ml)**

| **Grade** | **single score** | **Freshman and Sophomore** | **Junior and Senior** |
| --- | --- | --- | --- |
| excellent | **100** | 3400 | 3450 |
|  | **95** | 3350 | 3400 |
|  | **90** | 3300 | 3350 |
| good | **85** | 3150 | 3200 |
|  | **80** | 3000 | 3050 |
| Pass | **78** | 2900 | 2950 |
|  | **76** | 2800 | 2850 |
|  | **74** | 2700 | 2750 |
|  | **72** | 2600 | 2650 |
|  | **70** | 2500 | 2550 |
|  | **68** | 2400 | 2450 |
|  | **66** | 2300 | 2350 |
|  | **64** | 2200 | 2250 |
|  | **62** | 2100 | 2150 |
|  | **60** | 2000 | 2050 |
| failed | **50** | 1960 | 2010 |
|  | **40** | 1920 | 1970 |
|  | **30** | 1880 | 1930 |
|  | **20** | 1840 | 1890 |
|  | **10** | 1800 | 1850 |

**Table 1-5 Men's 50-meter running single item score table (s)**

| **Grade** | **single score** | **Freshman and Sophomore** | **Junior and Senior** |
| --- | --- | --- | --- |
| excellent | **100** | 6.7 | 6.6 |
|  | **95** | 6.8 | 6.7 |
|  | **90** | 6.9 | 6.8 |
| good | **85** | 7.0 | 6.9 |
|  | **80** | 7.1 | 7.0 |
| Pass | **78** | 7.3 | 7.2 |
|  | **76** | 7.5 | 7.4 |
|  | **74** | 7.7 | 7.6 |
|  | **72** | 7.9 | 7.8 |
|  | **70** | 8.1 | 8.0 |
|  | **68** | 8.3 | 8.2 |
|  | **66** | 8.5 | 8.4 |
|  | **64** | 8.7 | 8.6 |
|  | **62** | 8.9 | 8.8 |
|  | **60** | 9.1 | 9.0 |
| failed | **50** | 9.3 | 9.2 |
|  | **40** | 9.5 | 9.4 |
|  | **30** | 9.7 | 9.6 |
|  | **20** | 9.9 | 9.8 |
|  | **10** | 10.1 | 10.0 |

**Table 1-6 Femen's 50-meter running single item score table (s)**

| **Grade** | **single score** | **Freshman and Sophomore** | **Junior and Senior** |
| --- | --- | --- | --- |
| excellent | **100** | 7.5 | 7.4 |
|  | **95** | 7.6 | 7.5 |
|  | **90** | 7.7 | 7.6 |
| good | **85** | 8.0 | 7.9 |
|  | **80** | 8.3 | 8.2 |
| Pass | **78** | 8.5 | 8.4 |
|  | **76** | 8.7 | 8.6 |
|  | **74** | 8.9 | 8.8 |
|  | **72** | 9.1 | 9.0 |
|  | **70** | 9.3 | 9.2 |
|  | **68** | 9.5 | 9.4 |
|  | **66** | 9.7 | 9.6 |
|  | **64** | 9.9 | 9.8 |
|  | **62** | 10.1 | 10.0 |
|  | **60** | 10.3 | 10.2 |
| failed | **50** | 10.5 | 10.4 |
|  | **40** | 10.7 | 10.6 |
|  | **30** | 10.9 | 10.8 |
|  | **20** | 11.1 | 11.0 |
|  | **10** | 11.3 | 11.2 |

**Table 1-7 Single-item scoring table for male sitting forward flexion (cm)**

| **Grade** | **single score** | **Freshman and Sophomore** | **Junior and Senior** |
| --- | --- | --- | --- |
| excellent | **100** | 24.9 | 25.1 |
|  | **95** | 23.1 | 23.3 |
|  | **90** | 21.3 | 21.5 |
| good | **85** | 19.5 | 19.9 |
|  | **80** | 17.7 | 18.2 |
| Pass | **78** | 16.3 | 16.8 |
|  | **76** | 14.9 | 15.4 |
|  | **74** | 13.5 | 14.0 |
|  | **72** | 12.1 | 12.6 |
|  | **70** | 10.7 | 11.2 |
|  | **68** | 9.3 | 9.8 |
|  | **66** | 7.9 | 8.4 |
|  | **64** | 6.5 | 7.0 |
|  | **62** | 5.1 | 5.6 |
|  | **60** | 3.7 | 4.2 |
| failed | **50** | 2.7 | 3.2 |
|  | **40** | 1.7 | 2.2 |
|  | **30** | 0.7 | 1.2 |
|  | **20** | -0.3 | 0.2 |
|  | **10** | -1.3 | -0.8 |

**Table 1-8 Single-item scoring table for female sitting forward flexion (cm)**

| **Grade** | **single score** | **Freshman and Sophomore** | **Junior and Senior** |
| --- | --- | --- | --- |
| excellent | **100** | 25.8 | 26.3 |
|  | **95** | 24.0 | 24.4 |
|  | **90** | 22.2 | 22.4 |
| good | **85** | 20.6 | 21.0 |
|  | **80** | 19.0 | 19.5 |
| Pass | **78** | 17.7 | 18.2 |
|  | **76** | 16.4 | 16.9 |
|  | **74** | 15.1 | 15.6 |
|  | **72** | 13.8 | 14.3 |
|  | **70** | 12.5 | 13.0 |
|  | **68** | 11.2 | 11.7 |
|  | **66** | 9.9 | 10.4 |
|  | **64** | 8.6 | 9.1 |
|  | **62** | 7.3 | 7.8 |
|  | **60** | 6.0 | 6.5 |
| failed | **50** | 5.2 | 5.7 |
|  | **40** | 4.4 | 4.9 |
|  | **30** | 3.6 | 4.1 |
|  | **20** | 2.8 | 3.3 |
|  | **10** | 2.0 | 2.5 |

**Table 1-9 Standing long jump score sheet for male (cm)**

| **Grade** | **single score** | **Freshman and Sophomore** | **Junior and Senior** |
| --- | --- | --- | --- |
| excellent | **100** | 273 | 275 |
|  | **95** | 268 | 270 |
|  | **90** | 263 | 265 |
| good | **85** | 256 | 258 |
|  | **80** | 248 | 250 |
| Pass | **78** | 244 | 246 |
|  | **76** | 240 | 242 |
|  | **74** | 236 | 238 |
|  | **72** | 232 | 234 |
|  | **70** | 228 | 230 |
|  | **68** | 224 | 226 |
|  | **66** | 220 | 222 |
|  | **64** | 216 | 218 |
|  | **62** | 212 | 214 |
|  | **60** | 208 | 210 |
| failed | **50** | 203 | 205 |
|  | **40** | 198 | 200 |
|  | **30** | 193 | 195 |
|  | **20** | 188 | 190 |
|  | **10** | 183 | 185 |

**Table 1-10 Standing long jump score sheet for female (cm)**

| **Grade** | **single score** | **Freshman and Sophomore** | **Junior and Senior** |
| --- | --- | --- | --- |
| excellent | **100** | 207 | 208 |
|  | **95** | 201 | 202 |
|  | **90** | 195 | 196 |
| good | **85** | 188 | 189 |
|  | **80** | 181 | 182 |
| Pass | **78** | 178 | 179 |
|  | **76** | 175 | 176 |
|  | **74** | 172 | 173 |
|  | **72** | 169 | 170 |
|  | **70** | 166 | 167 |
|  | **68** | 163 | 164 |
|  | **66** | 160 | 161 |
|  | **64** | 157 | 158 |
|  | **62** | 154 | 155 |
|  | **60** | 151 | 152 |
| failed | **50** | 146 | 147 |
|  | **40** | 141 | 142 |
|  | **30** | 136 | 137 |
|  | **20** | 131 | 132 |
|  | **10** | 126 | 127 |

**Table 1-11 The single item score of male pull-up (times)**

| **Grade** | **single score** | **Freshman and Sophomore** | **Junior and Senior** |
| --- | --- | --- | --- |
| excellent | **100** | 19 | 20 |
|  | **95** | 18 | 19 |
|  | **90** | 17 | 18 |
| good | **85** | 16 | 17 |
|  | **80** | 15 | 16 |
| Pass | **78** |  |  |
|  | **76** | 14 | 15 |
|  | **74** |  |  |
|  | **72** | 13 | 14 |
|  | **70** |  |  |
|  | **68** | 12 | 13 |
|  | **66** |  |  |
|  | **64** | 11 | 12 |
|  | **62** |  |  |
|  | **60** | 10 | 11 |
| failed | **50** | 9 | 10 |
|  | **40** | 8 | 9 |
|  | **30** | 7 | 8 |
|  | **20** | 6 | 7 |
|  | **10** | 5 | 6 |

**Table 1-12 Scores of one-minute sit-ups for girls (times)**

| **Grade** | **single score** | **Freshman and Sophomore** | **Junior and Senior** |
| --- | --- | --- | --- |
| excellent | **100** | 56 | 57 |
|  | **95** | 54 | 55 |
|  | **90** | 52 | 53 |
| good | **85** | 49 | 50 |
|  | **80** | 46 | 47 |
| Pass | **78** | 44 | 45 |
|  | **76** | 42 | 43 |
|  | **74** | 40 | 41 |
|  | **72** | 38 | 39 |
|  | **70** | 36 | 37 |
|  | **68** | 34 | 35 |
|  | **66** | 32 | 33 |
|  | **64** | 30 | 31 |
|  | **62** | 28 | 29 |
|  | **60** | 26 | 27 |
| failed | **50** | 24 | 25 |
|  | **40** | 22 | 23 |
|  | **30** | 20 | 21 |
|  | **20** | 18 | 19 |
|  | **10** | 16 | 17 |

**Table 1-13 Male endurance running single item score table (minutes·seconds)**

| **Grade** | **single score** | **Freshman and Sophomore** | **Junior and Senior** |
| --- | --- | --- | --- |
| excellent | **100** | 3'17" | 3'15" |
|  | **95** | 3'22" | 3'20" |
|  | **90** | 3'27" | 3'25" |
| good | **85** | 3'34" | 3'32" |
|  | **80** | 3'42" | 3'40" |
| Pass | **78** | 3'47" | 3'45" |
|  | **76** | 3'52" | 3'50" |
|  | **74** | 3'57" | 3'55" |
|  | **72** | 4'02" | 4'00" |
|  | **70** | 4'07" | 4'05" |
|  | **68** | 4'12" | 4'10" |
|  | **66** | 4'17" | 4'15" |
|  | **64** | 4'22" | 4'20" |
|  | **62** | 4'27" | 4'25" |
|  | **60** | 4'32" | 4'30" |
| failed | **50** | 4'52" | 4'50" |
|  | **40** | 5'12" | 5'10" |
|  | **30** | 5'32" | 5'30" |
|  | **20** | 5'52" | 5'50" |
|  | **10** | 6'12" | 6'10" |

**Table 1-14 Female endurance running single item score table (minutes·seconds)**

| **Grade** | **single score** | **Freshman and Sophomore** | **Junior and Senior** |
| --- | --- | --- | --- |
| excellent | **100** | 3'18" | 3'16" |
|  | **95** | 3'24" | 3'22" |
|  | **90** | 3'30" | 3'28" |
| good | **85** | 3'37" | 3'35" |
|  | **80** | 3'44" | 3'42" |
| Pass | **78** | 3'49" | 3'47" |
|  | **76** | 3'54" | 3'52" |
|  | **74** | 3'59" | 3'57" |
|  | **72** | 4'04" | 4'02" |
|  | **70** | 4'09" | 4'07" |
|  | **68** | 4'14" | 4'12" |
|  | **66** | 4'19" | 4'17" |
|  | **64** | 4'24" | 4'22" |
|  | **62** | 4'29" | 4'27" |
|  | **60** | 4'34" | 4'32" |
| failed | **50** | 4'44" | 4'42" |
|  | **40** | 4'54" | 4'52" |
|  | **30** | 5'04" | 5'02" |
|  | **20** | 5'14" | 5'12" |
|  | **10** | 5'24" | 5'22" |

**Table 1-15 Scoring table for male pull-ups (times)**

| **Add points** | **Freshman and Sophomore** | **Junior and Senior** |
| --- | --- | --- |
| **10** | 10 | 10 |
| **9** | 9 | 9 |
| **8** | 8 | 8 |
| **7** | 7 | 7 |
| **6** | 6 | 6 |
| **5** | 5 | 5 |
| **4** | 4 | 4 |
| **3** | 3 | 3 |
| **2** | 2 | 2 |
| **1** | 1 | 1 |

**Table 1-16 Scores of one-minute sit-ups for female (times)**

| **Add points** | **Freshman and Sophomore** | **Junior and Senior** |
| --- | --- | --- |
| **10** | 13 | 13 |
| **9** | 12 | 12 |
| **8** | 11 | 11 |
| **7** | 10 | 10 |
| **6** | 9 | 9 |
| **5** | 8 | 8 |
| **4** | 7 | 7 |
| **3** | 6 | 6 |
| **2** | 4 | 4 |
| **1** | 2 | 2 |

Note: Pull-ups and one-minute sit-ups are high-quality indicators. After a student's score exceeds 100 points in a single item, the score corresponding to the number of times exceeded will be added.

**Table 1-17 Scoring table for the 1000-meter run for male (minutes·seconds)**

| **Add points** | **Freshman and Sophomore** | **Junior and Senior** |
| --- | --- | --- |
| **10** | -35" | -35" |
| **9** | -32" | -32" |
| **8** | -29" | -29" |
| **7** | -26" | -26" |
| **6** | -23" | -23" |
| **5** | -20" | -20" |
| **4** | -16" | -16" |
| **3** | -12" | -12" |
| **2** | -8" | -8" |
| **1** | -4" | -4" |

**Table 1-18 Scoring table for the 1000-meter run for female (minutes·seconds)**

| **Add points** | **Freshman and Sophomore** | **Junior and Senior** |
| --- | --- | --- |
| **10** | -50" | -50" |
| **9** | -45" | -45" |
| **8** | -40" | -40" |
| **7** | -35" | -35" |
| **6** | -30" | -30" |
| **5** | -25" | -25" |
| **4** | -20" | -20" |
| **3** | -15" | -15" |
| **2** | -10" | -10" |
| **1** | -5" | -5" |

Note: The 1000-meter run and the 800-meter run are all indicators of low excellence. After a student's score is lower than 100 points in a single item, the score corresponding to the number of seconds reduced will be added.
